# Supplementary material for: Dupilumab for the Treatment of Atopic Dermatitis in an Austrian Cohort-Real-Life Data Shows Rosacea-Like Folliculitis
Source: J Clin Med. 2020 Apr 24;9(4):1241. doi: 10.3390/jcm9041241 (PMC7230957; doi:10.3390/jcm9041241)
Supplement: Supplementary file 1 [file jcm-09-01241-s001.pdf]

Journal of
Clinical Medicine

MDPI

Supplementary material

Dupilumab for the Treatment of Atopic Dermatitis in an Austrian Cohort-Real-Life Data Shows Rosacea-Like Folliculitis

Tamara Quint [1], Patrick M. Brunner [1], Christoph Sinz [1], Irene Steiner [2], Robin Ristl [2], Kornelia Vigl [3], Detlev Pirkhammer [3], Martin Zikeli [4], Susanne Kimeswenger [5], Wolfram Hötzenecker [5], Katharina Neubauer [6], Norbert Reider [6] and Christine Bangert [1,*]

[1]Department of Dermatology, Medical University of Vienna, Vienna/Austria
[2] Institute of Medical Statistics, Medical University Vienna, Vienna/Austria
[3] Department of Dermatology, Rudolfstiftung City Hospital, Vienna/Austria
[4] Department of Dermatology, Wiener Neustadt Public Hospital, Wiener Neustadt/Austria
[5] Department of Dermatology, Kepler University Hospital, Linz/Austria
[6] Department of Dermatology, Medical University of Innsbruck, Innsbruck/Austria
* Correspondence: christine.bangert@meduniwien.ac.at; Tel.: +43-1-40400-77200

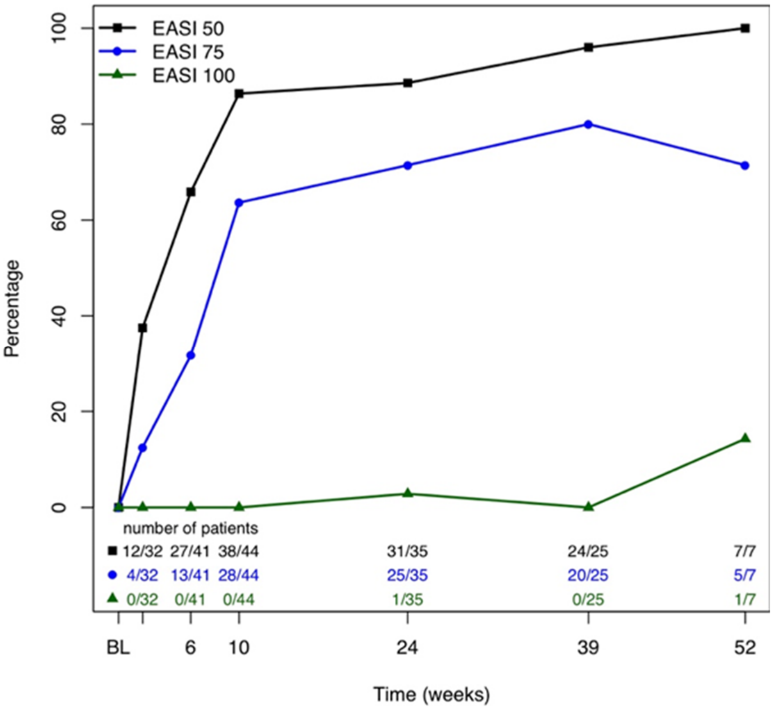

Figure S1. Effectiveness of dupilumab: EASI 50, 75 and 100.

J. Clin. Med. **2020**, *9*, 1241; doi:10.3390/jcm9041241 www.mdpi.com/journal/jcm
